# Adenomatoid odontogenic tumor associated with dentigerous cyst of the maxillary antrum: A rare entity

**DOI:** 10.4103/0973-029X.64308

**Published:** 2010

**Authors:** Simarpreet V Sandhu, Ramandeep S Narang, Manveen Jawanda, Sachin Rai

**Affiliations:** *Department of Oral and Maxillofacial Pathology, Genesis Institute of Dental Sciences and Research, Ferozepur, Punjab, India*; 1 *Department of Oral and Maxillofacial, Genesis Institute of Dental Sciences and Research, Ferozepur, Punjab, India*

**Keywords:** Adenomatoid odontogenic tumor, dentigerous cyst, maxillary antrum

## Abstract

Adenomatoid odontogenic tumor (AOT) is an uncommon tumor of odontogenic origin composed of odontogenic epithelium in a variety of histoarchitectural patterns. Most cases are in females and have a striking tendency to occur in the anterior maxilla. However, AOT of the maxillary antrum is extremely rare. A 25-year-old female presented with a large radiolucent lesion associated with the crown of an unerupted canine located in the maxillary antrum, which was clinically diagnosed as dentigerous cyst. The microscopic examination revealed the presence of AOT in the fibrous capsule of a dentigerous cyst. Very few cases of AOT associated with dentigerous cyst have been reported till date. A case of gigantic AOT that occupied the maxillary sinus and associated with dentigerous cyst is described. Also, an attempt has been made to determine whether the AOT derived from the dentigerous cyst could represent a distinct hybrid variety.

## INTRODUCTION

Adenomatoid odontogenic tumor (AOT) was first described by Ghosh[1] as an adamantinoma of the maxilla and was first recognized as a distinct pathological entity by Staphne[2] in 1948. According to the second edition of the WHO “Histological Typing of Odontogenic Tumors”,[3] AOT is defined as “A tumor of odontogenic epithelium with duct-like structures and with varying degrees of inductive change in the connective tissue. The tumor may be partly cystic, and in some cases the solid lesion may be present only as masses in the wall of a large cyst.”

AOT is an uncommon tumor of odontogenic origin, composed of odontogenic epithelium in a variety of histoarchitectural patterns. The lesion is benign (hamartomatous) and noninvasive, with slow but progressive growth. It accounts for 2–7% of all odontogenic tumors and is less frequent than odontoma, cementoma, myxoma and ameloblastoma. The majority of the cases (88%) are diagnosed in the second and third decades of life. The incidence is higher in males than in females (M:F–1:1.9). The tumor has a predilection for the anterior maxilla; however, AOT of maxillary antrum is extremely rare. There are only four cases reported so far.

The epithelial lining of the odontogenic cyst may transform into an odontogenic neoplasm-like ameloblastoma or AOT.[4–6] There have been many reports of odontogenic cysts associated with odontogenic tumors. The aim of this paper is to present a case of AOT that originated in the wall of a dentigerous cyst of the maxillary antrum, review the literature and stress that some AOTs can arise as a secondary phenomenon within the pre-existing dentigerous cysts.

## CASE REPORT

A 25-year-old female reported to the Department of Oral and Maxillofacial Surgery with the chief complaint of a swelling of the right cheek with right-sided nasal obstruction since 8 months. Intraoral examination revealed a firm well-defined swelling extending from the upper right central incisor to the second premolar of the same side. The swelling was nontender. The right upper cuspid was missing and 11, 12, 14 and 15 were vital. The overlying mucosa was nonulcerated and pink in color. There was no evidence of oro-nasal and oro-antral communication, and the palatal mucosa was intact.

**Figure 1:**
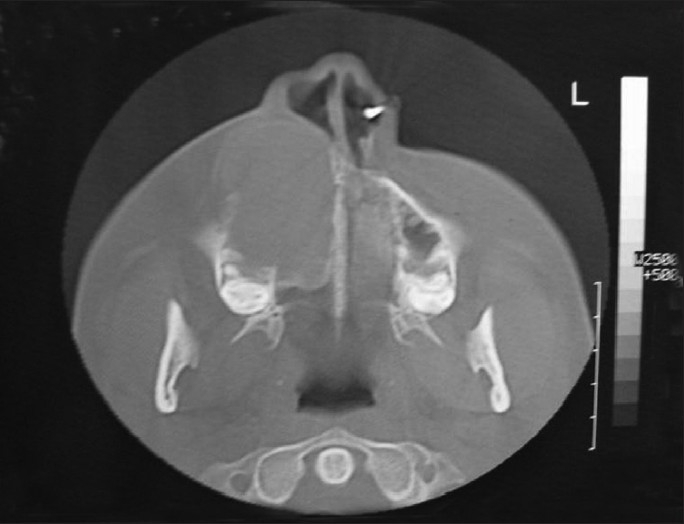
Axial computed tomography images showing expansion and thinning of the bony sinus wall that was absent at places

**Figure 2:**
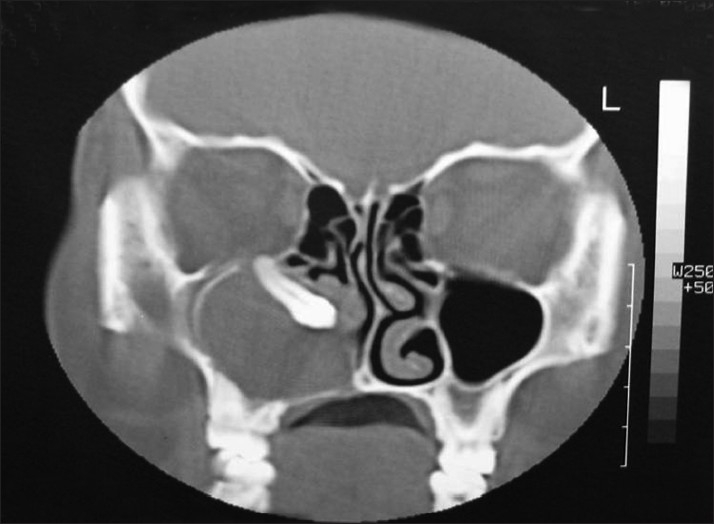
Coronal computed tomography images: an unerupted maxillary canine was seen near the mesial wall

**Figure 3:**
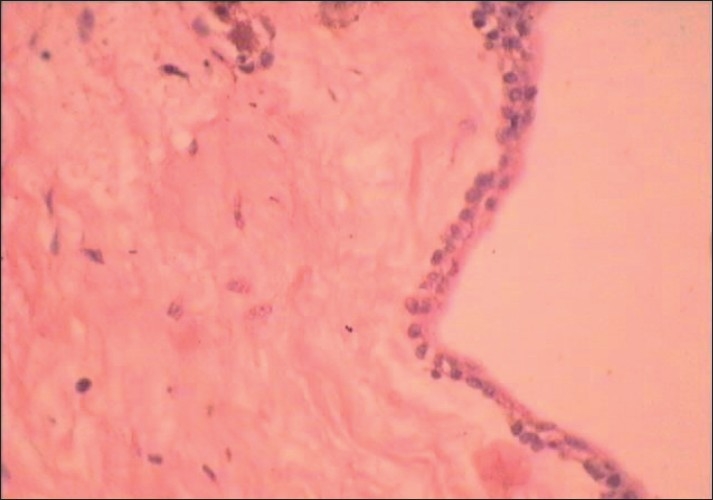
Photomicrograph showing reduced enamel epithelium two to three cell thick supported by loose bland connective tissue stroma (H and E, ×40).

**Figure 4:**
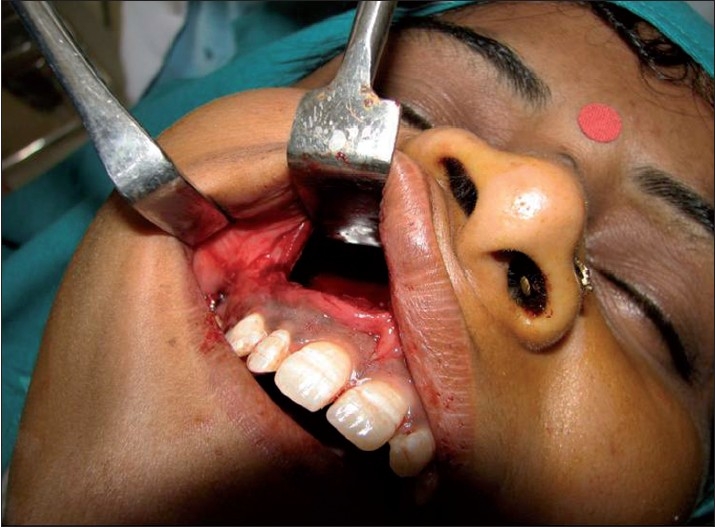
Picture showing the bony window within the portion of the labial plate

**Figure 5:**
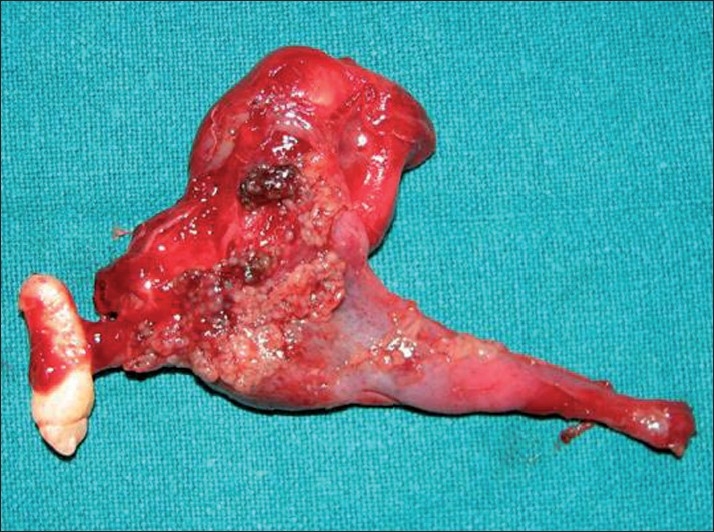
Enucleated specimen along with the embedded canine

**Figure 6:**
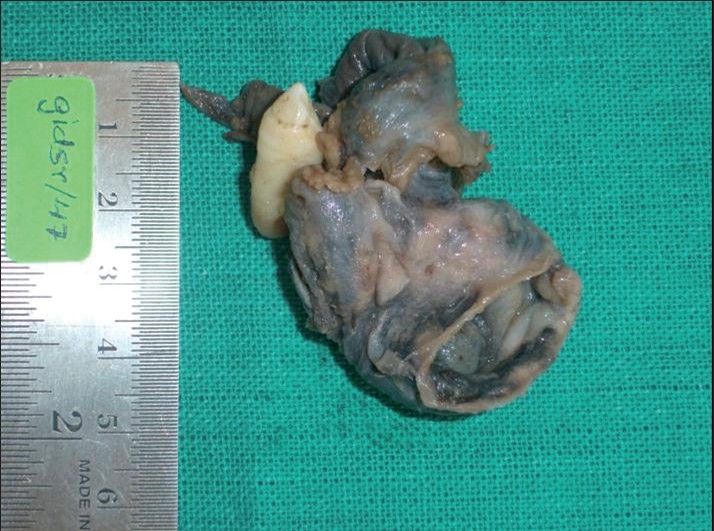
Gross examination revealed a cystic lesion measuring 6 cm × 5 cm × 4 cm

**Figure 7:**
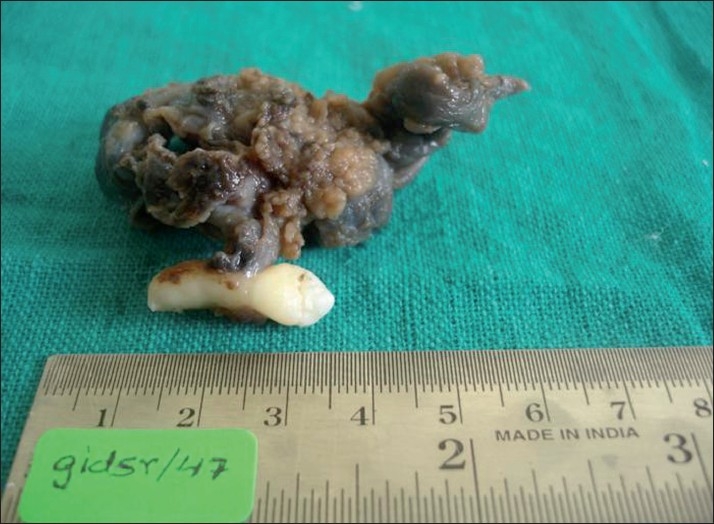
Gross specimen showing tan-colored thickenings in the wall of the cystic lesion

**Figure 8:**
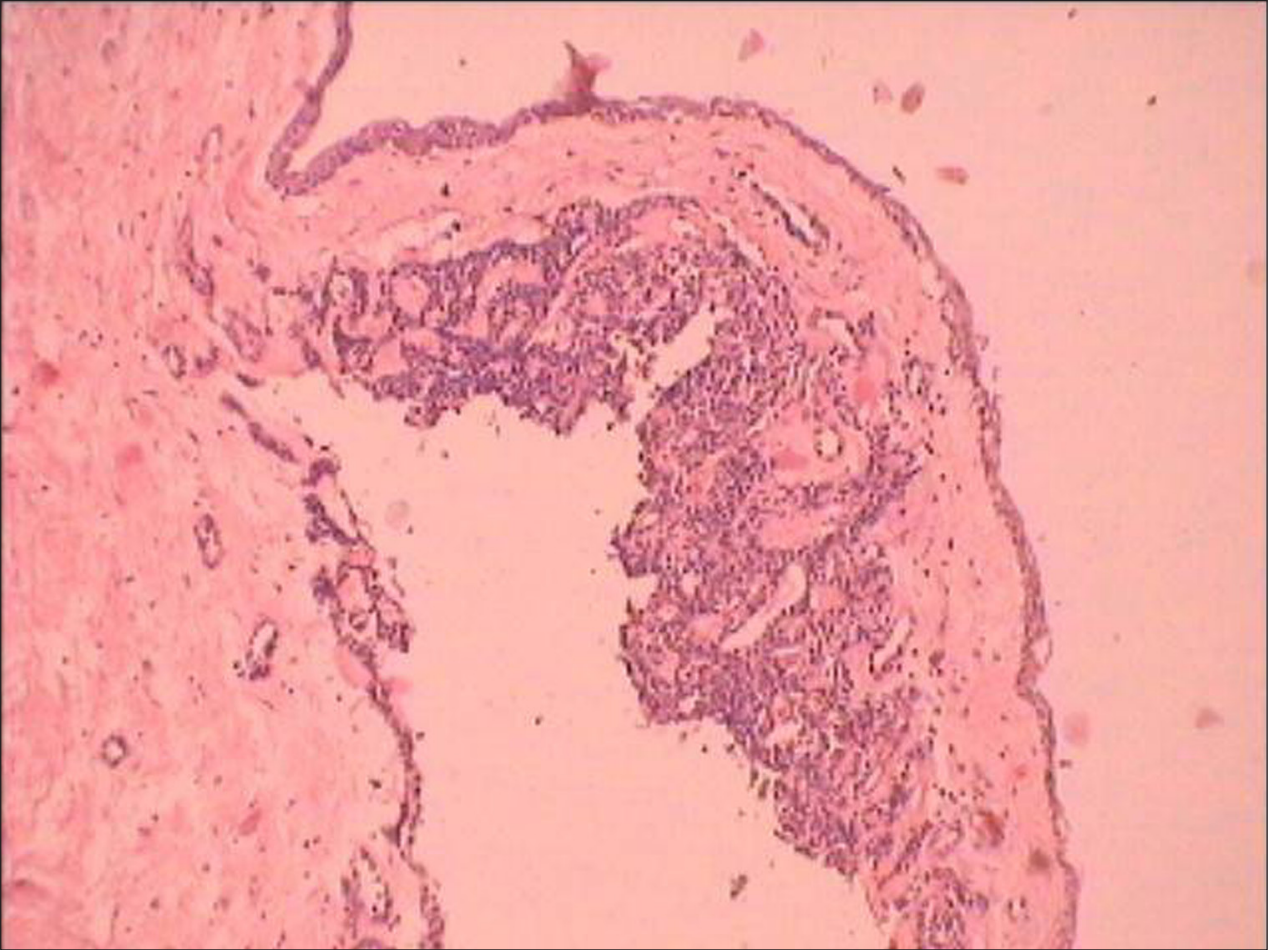
Photomicrograph showing the adenomatoid odontogenic tumor located in the fibrous capsule of the dentigerous cyst (H and E, ×10)

**Figure 9:**
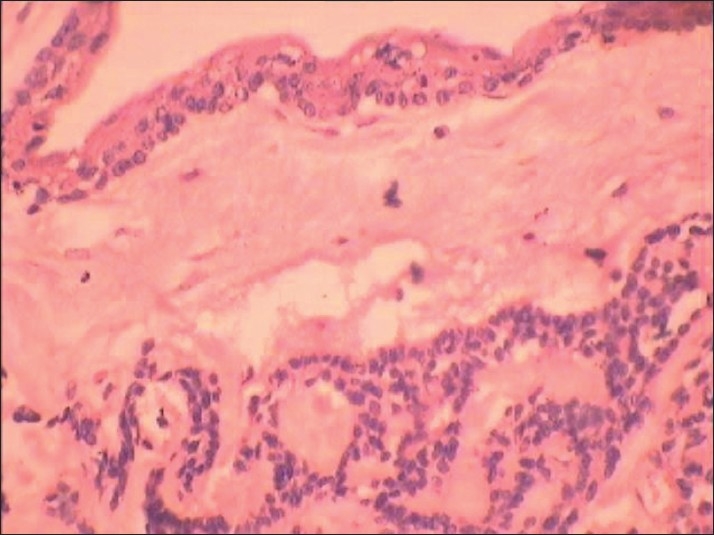
Photomicrograph showing the cribriform area showing cords of cells surrounding loose edematous connective tissue stroma (H and E, ×40)

**Figure 10:**
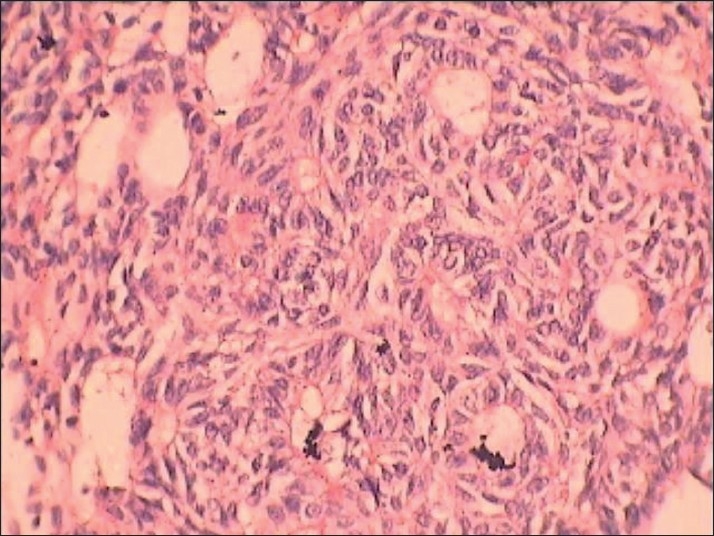
Photomicrograph showing the tubular structure enclosing a central space lined by columnar cells (H and E, ×40)

**Figure 11:**
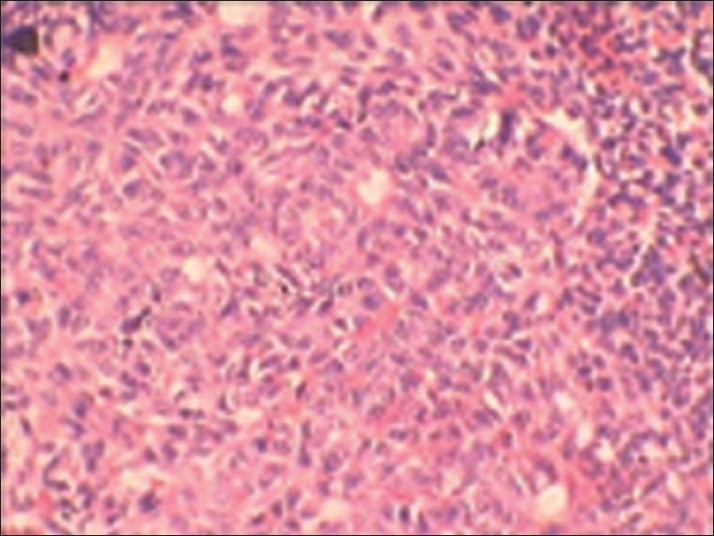
Photomicrograph showing cuboidal and columnar cells of the odontogenic epithelium forming rosette-like structures (H and E, ×40)

Computed tomography scan demonstrated a large lesion of the right maxillary sinus measuring 6 cm × 5 cm in dimension [Figure 1]. There was expansion and thinning of the bony sinus wall, which was absent at places. An unerupted maxillary canine was seen near the mesial wall [Figure 2]. Diagnostic aspiration was performed and about 15 ml of straw-colored fluid was aspirated. On the basis of the clinical and radiographic findings, the differential diagnosis of dentigerous cyst, AOT and odontogenic keratocyst was made. A small bony window of approximately 5 mm × 5 mm was made within the portion of labial plate that corresponded to the upper right central incisor. An incisional biopsy was performed and a histological diagnosis of dentigerous cyst with nonkeratinized epithelial lining and fibrous connective tissue was made [Figure 3]. The mass was enucleated completely along with the embedded canine and the specimen was submitted for histopathological examination [Figures 4 and 5]. Gross examination revealed a cystic lesion measuring 6 cm × 5 cm × 4 cm [Figure 6]. The cyst wall demonstrated tan-colored nodular thickenings in some areas [Figure 7]. Majority of the lesion comprised of reduced enamel epithelium two to three cell thick supported by a bland loose connective tissue stroma. Sections of the solid tissue containing histological characteristics of AOT were found in the fibrous capsule of the dentigerous cyst [Figure 8]. The tumor was composed of nodules of various sizes consisting of cuboidal or columnar epithelial cells that formed nests or rosette-like structures. In certain areas, cubical cells were arranged in a cribriform or lace-like pattern showing cords of cells with associated hyaline material surrounded by a loose oedematous vascular stroma [Figure 9]. This epithelium was continuous with the cuboidal or columnar cells of the odontogenic epithelium, forming nests or rosette-like structures [Figure 10]. In other areas, tubular structures enclosing a central space lined by columnar cells were also seen [Figure 11]. A diagnosis of AOT located in the fibrous capsule of the dentigerous cyst was made. The postoperative course was uneventful and, 6 months later, there were no signs of recurrence.

## DISCUSSION

AOT is composed of odontogenic epithelium in a variety of histoarchitectural patterns, embedded in a mature connective tissue stroma and characterized by slow but progressive growth. Although AOT is not as rare an odontogenic tumor, as had been previously thought, there have been few reports of the lesion located in the maxillary antrum that arose in the wall of the dentigerous cyst.

AOT was first recognized as a distinct pathological entity by Stafne in 1948.[2] There are three variants of AOT based on clinical and radiological features: the follicular type (accounting for 70.80% of the cases), which has a central lesion associated with an embedded tooth; the extrafollicular type (26.9% of the cases), which has a central lesion and no connection with the tooth; the peripheral variety (2.3% of the cases). Both types of central intraosseous tumors produce a corticated radiolucency, sometimes with radiopaque specks. The follicular type is usually initially diagnosed as a dentigerous or follicular cyst. The extrafollicular type usually presents as a unilocular, well-defined radiolucency found between, above or superimposed on the roots of the erupted teeth and often resembling a residual, radicular, globulomaxillary or lateral periodontal cyst. The peripheral type usually presents as a gingival swelling, located palatally or lingually relative to the involved tooth.

It has been reported that some odontogenic cysts occur in association with odontogenic tumors. Because neoplastic and hamartomatous lesions can occur at any stage of odontogenesis, odontogenic tumors with combined features of epithelial and mesenchymal components may arise within the odontogenic cyst.

In this case, AOT and dentigerous cyst are found in the same lesion. Clinical, radiographic and macroscopic findings in the present case are consistent with descriptions of the lesion in the dental literature. As previously mentioned, AOTs are usually solid but are occasionally cystic. Very few cases have been described that arise in association with a dentigerous cyst. A systematic search of the English language medical literature revealed only seven such cases, and only four cases of its occurrence in the maxillary sinus.

The structure of the cyst and its insertion around the crown of an unerupted tooth were typical of a dentigerous cyst. Odontogenesis is a complex process and neoplastic or hamartous lesions can occur at any stage of odontogenesis. The secondary development of an ameloblastic proliferation, whether hyperplastic or neoplastic, is well known but remains controversial. In this case, the multifocal cellular proliferation had the structure of an AOT. Its mural development in a dentigerous cyst is not uncommon. The tumor is benign and curettage is curative [Tables 1 and 2].

**Table 1:** Comparative clinical and radiologic features

Feature	D cyst	AOT	This case
Incidence	20% (jaw cyst)	3–7% (odontogenic tumor)	X
Age	10–30 (wide range)	10–19 (69%)	25
Gender	Male (slight)	Female (F:M=2:1)	Female
Site	Mandibular 8, Mx 3	Maxillary ant. (3)	Tooth 13
Symptom	Asymptomatic	Asymptomatic	Asymptomatic
X-ray	Unilocular R/L surrounding unerupted tooth	Unilocular R/L Unerupted tooth, 75% calcification, 33–66%	Unilocular R/L Impacted 13. No calcification

**Table 2:** Clinical data of the reported cases of adenomatoid odontogenic tumor arising from a dentigerous cyst

Reference	Age/Sex	Race	Radiographic	Features	Site
Valderrama[7]	16 Female	Philippino	Unilocular radiolucency	Tooth 14 crown surrounded	Maxilla
Warter *et al*.[8]	8 Male	Nigerian	Unilocular radiolucency	Tooth 13 crown surrounded	Maxilla
Tajima *et al*.[9]	15 Male	Japanese	A well-defined radiopaque mass	Crown of unerupted 28	Maxillary sinus
Garcia-Pola Vallejo *et al*.[4]	12 Male	Spanish	Unilocular radiolucency	Tooth 23 crown surrounded	Maxilla
Takahashi *et al*.[10]	22 Male	Japanese	Unilocular radiolucency	Tooth 28 crown surrounded	Maxilla
Bravo *et al*.[11]	14 Male	Not stated	Unilocular radiolucency	Tooth 23 crown surrounded	Maxilla
Chen *et al*.[12]	18 Male	Chinese	Unilocular radiolucency	Tooth 23 crown surrounded	Maxilla
Our case	25 Female	Indian	Unilocular swelling	Tooth 13 crown surrounded	Maxillary sinus

There is an uncertainty whether the lining of an associated cyst represents a true dentigerous cyst, cystic change within an AOT or may represent a distinct entity. Also, it is unclear whether this entity has a more aggressive potential. The AOT and dentigerous cyst are both benign, encapsulated lesions and conservative surgical enucleation or curettage is the treatment of choice. The prognosis for a dentigerous cyst is good and recurrences are very rare after complete removal of the lesion. There have been some rare reports of aggressive behavior on the part of AOT. As previously mentioned, AOTs are usually solid but may occasionally be cystic. Very few cases have been described that arise in association with a dentigerous cyst. Tajima *et al*.[9] describe an AOT located in the superior portion of the maxillary sinus and speculate that the tumor was derived from a dentigerous cyst. Philipsen *et al*.[13–15] also postulated that the follicular type of AOT develops from nests of cells within the dental lamina and, therefore, as a result, surrounds the tooth.

The hypothesis that follicular AOTs arise from the reduced enamel epithelium (REE) that lines the follicles of unerupted teeth is fairly conclusive and is supported by evidence that is both morphological and immunocytochemical in nature. They surround the crowns and are attached to the necks of unerupted teeth in a true follicular relationship. Many present as cystic lesions with only mural nodules of AOT lesional tissue and, in some instances, origin of the lesional tissue from the REE can be demonstrated histologically. Whether origin of the follicular variant occurs before or after cystic expansion has taken place is open to conjecture. If it occurs after cystic expansion, then this effectively means origin from a dentigerous cyst, and several such case reports have been published.[4,9,10] If it occurs before cystic expansion, then the tumor tissue will fill the follicular space and the AOT will present as a solid tumor. It is reasonable to assume that, given enough time, even those originating from a cyst may grow and fill the lumen completely. It cannot be ruled out that the dentigerous cyst with an impacted canine developed first followed by development of AOT in the cyst wall.

## CONCLUSION

Very few case reports of maxillary antrum AOT arising from a dentigerous cyst with histological identification have previously been reported. We believe that the present case represents an odontogenic cyst with neoplastic development, containing both epithelial and mesenchymal components. Meticulous histopathological evaluation is thus required of all enucleated cysts, which could contribute to the diagnosis of similar cases as reported in the present study.
